# Examining the Relationship Between Teachers’ ICT Self-Efficacy for Educational Purposes, Collegial Collaboration, Lack of Facilitation and the Use of ICT in Teaching Practice

**DOI:** 10.3389/fpsyg.2018.00935

**Published:** 2018-06-13

**Authors:** Ida K.R. Hatlevik, Ove E. Hatlevik

**Affiliations:** ^1^Department of Teacher Education and School Research, University of Oslo, Oslo, Norway; ^2^OsloMet – Oslo Metropolitan University, Oslo, Norway

**Keywords:** teachers, ICT self-efficacy for educational purposes, ICT self-efficacy, collegial collaboration, use of ICT, lack of facilitation

## Abstract

Information and communication technology (ICT) is now an integrated and central element of modern life, and its rapid emergence is changing the execution and organization of work and learning. Digital technology is also important for schools, and hence for teachers’ working days. However, among today’s teachers, not everyone has the knowledge required to teach using digital technology. Recent research indicates that self-efficacy is important for how teachers master their practice. This paper addresses teachers’ ICT self-efficacy for educational purposes, and examines the assumed antecedents of teachers’ self-efficacy. Data from 1,158 teachers at 116 Norwegian schools was analyzed. The results indicate that teachers’ self-efficacy for using ICT in their teaching practice is associated with their use of ICT in teaching and their general ICT self-efficacy. In addition, the results show that collegial collaboration among teachers has a positive association with the use of ICT in their teaching practice. One interpretation of these findings is that general ICT self-efficacy is necessary for developing ICT self-efficacy for educational purposes and being able to use ICT in education. However, further research is required to scrutinize the relationships between these concepts.

## Introduction

In little more than a generation, ICT has become a ubiquitous element of modern life. As schools prepare students to live in a technology-infused society and technology-driven workplaces, we must have teachers who are well prepared to support students’ learning through the use of technology. Yet, many of those teaching today came of age during a transitional time and have varying degrees of capacity and comfort with the array of technological tools at their disposal. Their capacity to enhance the learning of students with technology and to enhance their students’ technological skills depends, in part, on their personal comfort with and use of technological tools in their lives outside of the classroom. Beyond that, their motivation to infuse ICT in instruction instead of more traditional forms of pedagogy with which they may be more familiar is influenced by their belief in their capability to do so successfully. These self-efficacy beliefs regarding ICT instruction, as research in self-efficacy in other domains has demonstrated, are likely to influence the effort they invest in planning for and delivering ICT instruction, their persistence with students who struggle and their resilience in the face of the inevitable snafus and breakdowns that accompany any pedagogical innovation, and even more so an innovation involving the use of technology. Most previous studies have focused on the impact of one or two variables on either teachers’ self-efficacy in teaching or on their teaching practice. Thus, there is a need to gather knowledge about how different variables interact and are associated with both teachers’ ICT self-efficacy for instructional purposes and with the use of ICT in their teaching practice. In this article, we explore the associations between teachers’ self-efficacy in using ICT for instructional purposes, the use of ICT in teaching practice, general ICT self-efficacy, collegial collaboration regarding the use of ICT in teaching and the lack of facilitation for using ICT in teaching by the school management. The analysis is based on Norwegian teachers’ answers to questions in the International Computer and Information Literacy study (ICILS) 2013 (Fraillon et al., 2014). In the following two sections, we elaborate on the studies used to formulate the hypotheses tested in this article.

### Self-Efficacy

Bandura’s (1997, p. 3) concept of “self-efficacy refers to a belief in one’s capabilities to organize and execute the courses of action required to produce given attainments.” For the teaching profession, “a teacher’s efficacy belief is a judgment of his or her capabilities to bring about desired outcomes of student engagement and learning, even among those students who may be difficult or unmotivated” (Tschannen-Moran and Hoy, 2001, p. 783). In other words, teacher self-efficacy is about “teachers’ beliefs that they are capable of carrying out good teaching in the classroom” (Christophersen et al., 2016, p. 241).

Previous research has underscored the fact that teachers’ self-efficacy has an effect on their job satisfaction and professional commitment (Skaalvik and Skaalvik, 2007; Ware and Kitsantas, 2007), attrition from the teaching profession (Klassen and Chiu, 2011; Hong, 2012) and is an important predictor of students’ motivation and achievements (Caprara et al., 2006; Guo et al., 2012). Thus, identifying factors that can influence teachers’ self-efficacy in using ICT in their teaching practice is an important subject to investigate. Social cognitive theory points to a potential positive effect of individuals’ perception of their own competence and capabilities in a specific area of interest (i.e., self-efficacy) for continual growth and a feeling of mastery in that same field and similar fields of interest. Bandura (1997) claimed that these beliefs were more powerful than one’s actual abilities; thus, self-efficacy beliefs can become self-fulfilling prophesies. Bandura stated that self-efficacy in a specific area affects individuals’ thought processes, levels of persistence, degrees of motivation and affective states regarding tasks within the same area, thereby influencing individuals’ performances. Enhancing individuals’ self-efficacy beliefs in a specific set of tasks increases their performance levels on those tasks; however, those same individuals may fail in tasks that exceed their perceived coping capabilities (Bandura, 1997). Recent research regarding self-efficacy and the use of ICT in teaching corroborates Bandura’s assumptions, and underscores the notion that increased levels of computer self-efficacy can lead to higher levels of confidence in being an efficient teacher with ICT (Fanni et al., 2013). Hammond et al. (2011) examined reasons why teachers use ICT, and they discovered a relationship between lower levels of ICT self-efficacy and the less frequent use of ICT. Furthermore, recent research demonstrates a positive relationship between self-efficacy about using digital tools and the use of ICT for teaching purposes (Teo, 2014; Hatlevik, 2017). In addition there is a positive association between student teachers’ use of computers and their computer self-efficacy (So et al., 2012).

According to Bandura (1997), self-efficacy is both domain and context specific (i.e., it is not a global trait). In this study, we focus on teachers’ ICT self-efficacy for instructional purposes, which describes the self-confidence teachers have when it comes to using ICT in their own teaching and instruction (Krumsvik, 2014). Krumsvik (2011) distinguishes between being confident about using ICT on your own and using ICT for pedagogical purposes. Scherer and Siddiq (2015), who used the same data as we utilize in our study, also reported that computer self-efficacy in basic and advanced ICT operational and collaborative skills, and self-efficacy in using computers for instructional purposes, are highly correlated but separate constructs. One way to interpret this positive association is that teachers’ general perception of their own ICT skills (general ICT self-efficacy) is a necessary, but not a sufficient, determinant for self-efficacy in using ICT for instructional purposes. This interpretation makes sense, as you need to be competent in a skill yourself in order to be able to incorporate it when instructing others. A reasonable assumption to draw from Bandura’s (1997) theory of self-efficacy and the results of the various studies mentioned here is that teachers’ ICT self-efficacy for instructional purposes is positively related to their general ICT self-efficacy (hypothesis 1, **Table 1**), and to their use of ICT in teaching practice (hypothesis 2, **Table 1**).

**Table 1:** Hypothesized relations between teachers’ ICT self-efficacy for instructional purposes, use of ICT, general ICT self-efficacy, collegial collaboration and lack of facilitation.

Hypothesis 1 (H1)	Teachers’ general ICT self-efficacy has a positive association with their ICT self-efficacy for instructional purposes.
Hypothesis 2 (H2)	Teachers’ ICT self-efficacy for instructional purposes has a positive association with teachers’ use of ICT in teaching practice.
Hypothesis 3 (H3)	Collegial collaboration has a positive association with teachers’ ICT self-efficacy for instructional purposes.
Hypothesis 4 (H4)	Collegial collaboration has a positive association with teachers’ use of ICT in their teaching practice.
Hypothesis 5 (H5)	Lack of facilitation for using ICT by the school management has a negative association with teachers’ ICT self-efficacy for instructional purposes.
Hypothesis 6 (H6)	Lack of facilitation for using ICT in teaching by the school management has a negative association with teachers’ use of ICT in teaching practice.


Bandura (1997) asserted that there are four major influences on self-efficacy beliefs – vicarious experiences, verbal persuasion, physiological arousal and mastery experiences. In our study, we focus on how general ICT self-efficacy and contextual factors like collegial collaboration regarding the use of ICT in teaching, and the lack of facilitation for using ICT in teaching by the school management, are associated with ICT self-efficacy for instructional purposes. One can argue that collegial collaboration in particular entails the opportunity for both vicarious experiences and verbal support and persuasion. Furthermore, a lack of facilitation by the management could be interpreted as a hindrance for developing ICT self-efficacy for instructional purposes. In the next section, we elaborate on research related to the relations between contextual factors and teachers’ ICT self-efficacy for instructional purposes and the actual use of ICT in teaching.

### Contextual Factors: Collaboration and Facilitation

The results from the Teaching and Learning International Survey (TALIS) 2013 show that Norwegian teachers are requesting assistance to develop their professional digital competence (Organisation for Economic Co-operation and Development [OECD], 2014). Previous studies have identified an association between facilitating teachers’ use of ICT and their professional digital literacy development (Krumsvik, 2011; Tondeur et al., 2012). Tondeur et al. (2012) emphasize that learning from peers and collegial collaboration are productive ways for pre-service teachers to learn how to implement ICT in their teaching practice. Furthermore, findings from a research project including teachers from all the EU countries (Wastiau et al., 2013), indicate that teachers prefer an informal approach to learn how to use ICT. Teachers do not seem to prefer external courses when developing their digital competence (Egeberg et al., 2011). Recent research indicates that teachers want to learn about ICT together with other teachers (Bacigagulpo and Cachia, 2011) and participate in training activities related to authentic classroom settings (Balanskat et al., 2006). One way to interpret these research findings is that collegial collaboration provides informal opportunities for teachers to learn about ICT together with other teachers in order to foster ICT self-efficacy and understand how to use ICT for educational purposes. This notion is also supported by previous research, which has shown that teachers’ professional self-efficacy is positively affected by interpersonal support (Tschannen-Moran and Hoy, 2007) and collegial collaboration (Goddard et al., 2007), collective work, cooperation and exchanges amongst teachers (Grangeat and Gray, 2008). Furthermore, Caspersen and Raaen (2014) identify both collegial and superiors’ support as influential in terms of teachers’ perceived mastery of teaching. Finally, previous research indicates that collegial collaboration is of importance when it comes to teachers’ actual teaching practice and students’ achievements (Goddard et al., 2007).

Thus, taken together, these findings indicate that contextual factors like collegial collaboration are positively associated with teachers’ ICT self-efficacy for instructional purposes (hypothesis 3, **Table 1**) and their actual teaching practice (hypothesis 4, **Table 1**). Furthermore, we expect support and facilitation by the school management to be associated with teachers’ ICT self-efficacy for instructional purposes and their actual use of ICT in teaching practice. However, the data in our study focuses on the lack of facilitation by the school management, which we expect to be negatively associated with ICT self-efficacy for instructional purposes (hypothesis 5, **Table 1**) and the use of ICT in teaching practice (hypothesis 6, **Table 1**).

### Aim of the Present Study

The aim of this paper is to investigate the relationship between teachers’ self-efficacy in using ICT for instructional purposes, the use of ICT in teaching practice, general ICT self-efficacy, collegial collaboration regarding the use of ICT in teaching and the lack of facilitation for using ICT in teaching by the school management. Six hypotheses regarding the relationships between these variables were developed from the existing research literature.

Two hypotheses address to what extent general ICT self-efficacy relates to ICT self-efficacy for instructional purposes (H1, **Table 1**) and to the reported use of ICT in teaching practice (H2). Furthermore, two hypotheses address the extent to which teachers’ perception of collegial collaboration relates to ICT self-efficacy for instructional purposes (H3) and to their reported use of ICT in teaching practice (H4). Finally, two hypotheses address the association between the reported lack of facilitation for ICT self-efficacy for instructional purposes (H5) and their reported use of ICT in teaching practice (H6). The hypotheses tested in this paper are presented in **Table 1**.

**FIGURE 1:**
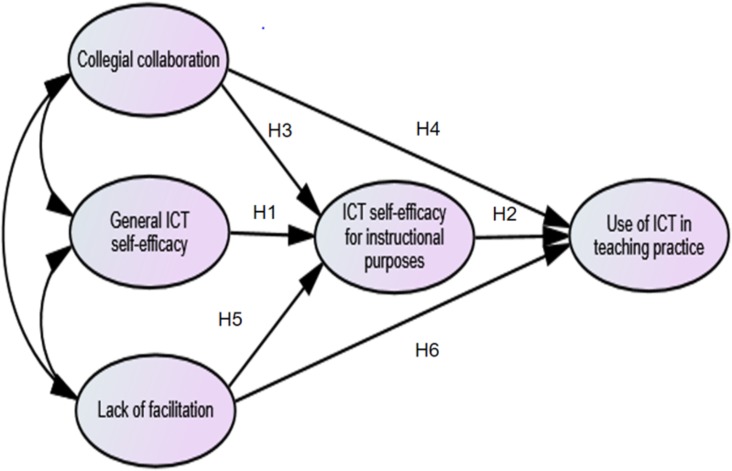
Theoretical model of the relationship between the variables expected to be associated with teachers’ ICT self-efficacy for instructional purposes and their use of ICT in teaching practice.

**Figure 1** shows the hypothesized relationships between the variables, indicating that teachers’ ICT self-efficacy for instructional purposes acts as a mediating variable.

## Materials and Methods

### Participants and Procedure

This study has a cross-sectional, correlational design, and is a secondary analysis of existing data, namely the Norwegian ICILS 2013 consisting of responses from 1158 secondary schoolteachers. The International Association for the Evaluation of Educational Achievement (IEA^1^) (Fraillon et al., 2014) conducted the collection, coding and reporting of the data according to predefined quality standards. The study had a two-step design. First, 150 schools were randomly selected. Second, based on the size of the school and the number of teachers in the school, between 15 and 20 teachers were selected from each school. Respondents from 116 schools replied to the survey (79% response rate at school level). The sample consisted of 64% female teachers. The ages of the respondents were measured in six intervals; 2% were younger than 25 years old, 9% were between 25 and 30, 30% were between 30 and 39, 27% were between 40 and 49, 19% were between 50 and 59 and 13% were 60 or older.

### Instruments

The teachers answered an online questionnaire that contained questions and statements about their ICT self-efficacy, the use of ICT in teaching and contextual factors. All the questions and statements used in the analysis are presented in **Table 2**, along with information about descriptive statistics and univariate normality, and the factor loadings obtained from the latent variable models for the scales.

**Table 2:** Means, standard deviations, skewness, kurtosis, and factor loadings for all items of the administered scales.

Scale Items	*M* (*SD*)	Skewness	Kurtosis	Standardised factor loadings (*SE*)
**Use of ICT for the following practices (Cronbach’s α = 0.79)**			
Presenting information through direct class instruction	2.34 (0.56)	-0.10	-0.73	0.52 (0.04)^∗∗^
Providing remedial or enrichment support	1.93 (0.58)	0.01	-0.09	0.55 (0.03)^∗∗^
Enabling student-led whole-class discussions and presentations	1.76 (0.62)	0.21	-0.59	0.57 (0.03)^∗∗^
Assessing students’ learning through tests	1.87 (0.65)	0.13	-0.66	0.62 (0.03)^∗∗^
Providing feedback to students	2.11 (0.70)	-0.16	-0.96	0.61 (0.04)^∗∗^
Reinforcing the learning of skills through repetition of examples	1.90 (0.58)	0.01	-0.12	0.63 (0.03)^∗∗^
Supporting collaboration among students	1.62 (0.61)	0.42	-0.66	0.63 (0.04)^∗∗^
**Collegial collaboration when using ICT in teaching and learning (Cronbach’s α = 0.71)**			
I work together with other teachers	2.50 (0.71)	-0.05	-0.25	0.72 (0.03)^∗∗^
I systematically collaborate with colleagues to develop ICT-based lessons	2.16 (0.70)	0.48	0.45	0.72 (0.04)^∗∗^
**General ICT self-efficacy: How well can you… (Cronbach’s α = 0.75)**			
Use a spreadsheet program for keeping records or analyzing data	2.35 (0.73)	-0.64	-0.89	0.54 (0.03)^∗∗^
Contribute to a discussion forum/user group on the Internet	2.42 (0.67)	-0.72	-0.58	0.65 (0.04)^∗∗^
Collaborate with others using shared resources such as [Google Docs]	2.23 (0.66)	-0.28	-0.75	0.72 (0.04)^∗∗^
Install software	2.44 (0.72)	-0.90	-0.56	0.64 (0.04)^∗∗^
**ICT self-efficacy for instructional purposes: How well can you… (Cronbach’s α = 0.68)**			
Monitor students’ progress	1.71 (0.45)	-0.95	-1.11	0.86 (0.03)^∗∗^
Prepare lessons that involve the use of ICT by students	1.90 (0.30)	-2.62	4.87	0.77 (0.05)^∗∗^
Assess student learning	1.78 (0.42)	-1.33	-0.23	0.89 (0.03)^∗∗^
**Lack of facilitation by the school management (Cronbach’s α = 0.74)**			
There is not sufficient time to prepare lessons that incorporate ICT	2.55 (0.78)	0.13	-0.44	0.64 (0.04)^∗∗^
There is not sufficient provision for me to develop expertise in ICT	2.62 (0.77)	0.10	-0.48	0.92 (0.06)^∗∗^
There is not sufficient technical support to maintain ICT resources	2.59 (0.84)	0.10	-0.66	0.56 (0.04)^∗∗^


*^∗∗^*p* < 0.01.*

#### Use of ICT at School

Seven questions asked about the extent to which teachers use ICT in their teaching (e.g., presenting information through direct class instruction and supporting collaboration among students). The corresponding response categories were: 1 = Never, 2 = Sometimes and 3 = Often.

#### Collegial Collaboration When Using ICT in Teaching and Learning

Collegial collaboration when using ICT in teaching and learning was measured with two statements about whether or not they work together with other teachers and collaborate with their colleagues in developing ICT-based lessons. The corresponding response categories were: 1 = strongly disagree, 2 = disagree, 3 = agree and 4 = strongly agree.

#### General ICT Self-Efficacy

Four questions related to teachers’ beliefs in their capabilities to use ICT to perform certain general tasks on the computer (using a spreadsheet program, contributing to a discussion forum, collaborating with others and installing software). The corresponding response categories were: 1 = I do not think I could do this, 2 = I could work out how to do this and 3 = I know how to do this.

#### ICT Self-Efficacy for Instructional Purposes

This concept was measured with three questions about how well they could carry out specific tasks using ICT related to their teaching practice (monitoring students’ progress, preparing lessons that involve the use of ICT by students and assessing student learning). The corresponding response categories were: 1 = strongly disagree, 2 = disagree, 3 = agree and 4 = strongly agree.

#### Lack of Facilitation

Lack of facilitation was measured through three negative statements about whether or not they experienced a lack of facilitation and support from the school management in using ICT in teaching (insufficient preparation time, provisions to develop expertise and technical support). The corresponding response categories were: 1 = strongly disagree, 2 = disagree, 3 = agree and 4 = strongly agree.

### Analytical Strategy

Prior to testing the hypothesized model, the data was analyzed with respect to its descriptive statistics (means and standard deviations) and measures of univariate normality (skewness and kurtosis). Structural equation modeling (SEM) was used to test the assumed relationship between the variables (statistical software package Mplus 7.11). Such SEM allows for testing patterns of associations between latent variables, and at the same time can incorporate a measurement model that represents observed variables as indicators of underlying factors (Kline, 2010). Furthermore, SEM provides information that can be used to discuss how well the hypothesized model fits the empirical data (Brown, 2006). The model tested in this paper is a fully latent model; that is, the model examines the relationships amongst five latent variables (see **Figure 1**).

In order to evaluate the goodness-of-fit of the model (**Figure 1**), we used the chi-square information, together with the Comparative Fit Index (CFI), the Tucker-Lewis Index (TLI) and the Root Mean Square Error of Approximation (RMSEA) (Brown, 2006; Kline, 2010). The Weighted Root Mean Square Residual (WRMR) was used (Yu, 2002), because one latent dependent variable consist of categorical data. When evaluating the information from the fit indices, we are following guidelines recommended in the literature (Hu and Bentler, 1999; Marsh et al., 2004). A good model fit can be described with levels of the CFI and TLI equal to or above 0.95 (Marsh et al., 2004), RMSEA below or equal to 0.08 (Hu and Bentler, 1999), and levels of WRMR close to or below 1.00 (Yu, 2002). There are missing values for some items, and the full-information-maximum procedure was therefore used.

## Results

### Descriptive Statistics

The values for mean, standard deviation, skewness and kurtosis are presented in **Table 2**. The levels of skewness and kurtosis were acceptable for the items used to measure the use of ICT in teaching, collegial collaboration, lack of facilitation and general ICT self-efficacy. One item (preparing lessons that involve the use of ICT by students) used to measure ICT self-efficacy for instructional purposes had higher levels of both skewness and kurtosis. All items used to measure ICT self-efficacy for instructional purposes are therefore treated as categorical data in the analyses. The responses were recoded under two categories: 1 = I do not know how to do it or 2 = I do know how to do it. The first category includes responses to both original ratings of 1 (I do not think I could do this) and 2 (I could work out how to do this). This is the appropriate way to conduct analysis when data is not normally distributed.

### Measurement Model

The computed chi-square value of the tested model is significant (*p* = 0.000). However, the chi-square test is sensitive to large samples, and the current sample consists of 1158 respondents. The results of the other fit indices indicate a good model fit: CFI = 0.964, TLI = 0.958, RMSEA = 0.034 (90% CI = 0.029–0.039) and WRMR = 0.977.

Item loadings can be used to examine how the items reflect the constructs. Item loadings above 0.60 are desirable, but items with lower loadings can also provide relevant information about the constructs. Most of the factor loadings of each latent variable were relatively high (range = 0.52–0.92), indicating sufficient convergent validity (see **Table 2**). Cronbach’s alpha reliability coefficients for the respective latent variables were 0.79 for use of ICT in teaching practice (seven items), 0.71 for collegial support and cooperation (three items), 0.75 for general ICT self-efficacy (four items), 0.68 for ICT self-efficacy for instructional purposes (three items) and 0.74 for lack of facilitation for using ICT in teaching by the school management (three items). Overall, it seems that most items are working quite well.

The correlation matrix (**Table 3**) shows significant correlations between all the latent variables. There are positive moderate to high correlations between collegial collaboration, general ICT self-efficacy, ICT self-efficacy for instructional purposes and ICT use. Lack of facilitation correlates negatively with all of the other latent variables.

**Table 3:** Correlation matrix for all constructs.

Variables	1	2	3	4	5
(1) Collegial collaboration	–				
(2) Lack of facilitation	-0.337^∗∗^	–			
(3) General ICT self-efficacy	0.295^∗∗^	-0.115^∗∗^	–		
(4) ICT self-efficacy for instructional purposes	0.307^∗∗^	-0.141^∗∗^	0.715^∗∗^	–	
(5) ICT use	0.453^∗∗^	-0.110^∗^	0.341^∗∗^	0.509^∗∗^	–


*^∗^*p* < 0.05, ^∗∗^*p* < 0.01.*

**FIGURE 2:**
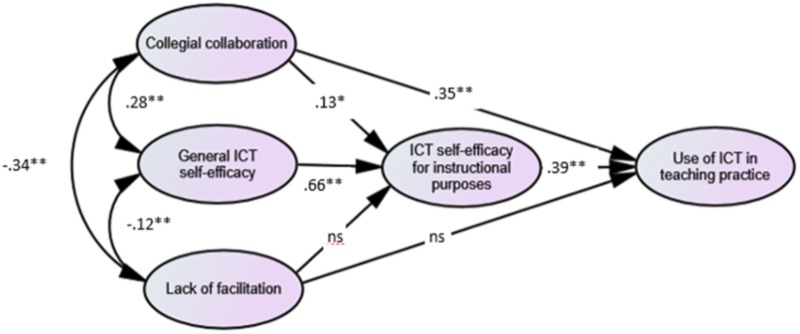
Standardized estimates for the SEM analysis of the relationship between the variables expected to have an effect on teachers’ ICT self-efficacy for instructional purposes and their use of ICT in teaching practice. Fit indices: Chi-square = 331.150, df = 143, and *p* = 0.0000; CFI = 0.964; TLI = 0.958; RMSEA = 0.034 (90% CI = 0.029–0.039); and WRMR = 0.0977. ^∗^*p* < 0.05, ^∗∗^*p* < 0.01; ns, not significant.

The results of the SEM analysis presented in **Figure 2** indicate that teachers’ general ICT self-efficacy has a strong positive association (β = 0.66) with teachers’ ICT self-efficacy for instructional purposes, thus corroborating hypothesis 1. In addition, collegial collaboration is positively associated (β = 0.13) with ICT self-efficacy for instructional purposes, thus corroborating hypothesis 3. Furthermore, teachers’ ICT self-efficacy for instructional purposes (β = 0.39) and collegial collaboration (β = 0.35) have moderate positive associations with teachers’ use of ICT in teaching practice, thus corroborating hypotheses 2 and 4.

Lack of facilitation for using ICT in teaching by the school management does not have a significant direct association with either teachers’ ICT self-efficacy for instructional purposes or with their use of ICT in teaching. However, there are moderate correlations between the three independent variables: teachers’ general ICT self-efficacy, collegial collaboration and lack of facilitation. Lack of facilitation correlates negatively with both collegial collaboration and general ICT self-efficacy, whereas collegial collaboration and general ICT self-efficacy are positively correlated.

Explained variance is 50.1% for teachers’ ICT self-efficacy for instructional purposes and 33.7% for teachers’ use of ICT in teaching practice.

An alternative model was tested, in which a direct association between the variables general ICT self-efficacy and use of ICT in teaching practice was added to the original model. This specific association came out as not significant, and the rest of the associations in the model did not change.

## Discussion

Previous research findings show variation when it comes to how teachers are able to use ICT efficiently in their own teaching practice (Fraillon et al., 2014; Haydn, 2014; Hatlevik, 2017). In addition, previous studies indicate that formal teaching competence alone is not a sufficient factor for effective student learning, as other individual and contextual factors are also influential (Gustafsson, 2003; Hattie, 2008). However, some individual characteristics seem to be more vital than others for good teaching practice; in particular, teachers’ self-efficacy in teaching is considered a key issue (Bandura, 1997; Skaalvik and Skaalvik, 2007).

This paper addresses a model with antecedents of teachers’ ICT self-efficacy for instructional purposes. A model was developed based on recent research regarding the relationship between collegial collaboration, general ICT self-efficacy, lack of facilitation, ICT self-efficacy for instructional purposes and the use of ICT in teaching practice. This assumed model consists of six hypotheses, and our analyses indicate that four of these six hypotheses are supported by the data.

First, teachers’ general ICT self-efficacy has a strong positive association with ICT self-efficacy for instructional purposes. This finding is in line with a fundamental premise within self-efficacy theory (Bandura, 1997) about the importance of distinguishing between domain-specific self-efficacy beliefs. Furthermore, the results show the importance of associating a domain-specific ICT self-efficacy with different ICT tasks or activities, e.g., ICT self-efficacy for instructional purposes. This finding seems to nuance the relationship between various types of domain- or task-specific self-efficacy, as the finding underpins the fact that the two constructs (general ICT self-efficacy and ICT self-efficacy for instructional purposes) are both distinctive concepts and highly correlated (Krumsvik, 2011; Scherer and Siddiq, 2015).

Second, teachers’ ICT self-efficacy for instructional purposes has a moderate positive association with their use of ICT, which corroborates previous findings that teachers’ digital competence predicts their use of ICT in their teaching practice (Krumsvik, 2011; Hatlevik, 2017). However, our analysis extends previous knowledge about the nature of the relationship between the use of ICT in teaching practice, ICT self-efficacy for instructional purposes and general ICT self-efficacy. Our results reveal how ICT self-efficacy for instructional purposes can act as a mediating variable. Thus, it is not enough to be confident in using ICT yourself (general perception of your own ICT skills); you also need to be confident about how to use it for instructional purposes. Therefore, supporting prospective and more experienced schoolteachers’ development of didactical competence in using ICT for instructional purposes is crucial when it comes to implementing ICT in teaching practice.

Third, collegial collaboration has a positive association with teachers’ use of ICT in their teaching practice, their general ICT self-efficacy and ICT self-efficacy for instructional purposes. Our results are in line with previous research, which underlines that teachers’ self-efficacy and their teaching practice is positively affected by collegial collaboration (Goddard et al., 2007; Caspersen and Raaen, 2014). Bandura (1997) emphasizes the social aspect of self-efficacy, meaning that self-efficacy is developed and influenced by the context of the person.

Fourth, the hypotheses (H5 and H6) regarding a lack of facilitation are not supported by the data. Based on the theory of Bandura (2006), we assumed that facilitation is important for self-efficacy. One explanation could be that there is a difference between the need for collaboration and facilitation between various groups of teachers. Thus, it would be interesting to test the model on both newly qualified and more experienced teachers, and on teachers from different countries.

Overall, there are some limitations to our study. First, the data is gathered from a cross-sectional design and it does not establish which factor comes first, meaning that it is difficult to show what is the cause and what is the effect. According to social cognitive theory, there are reasons to believe that a reciprocal relationship exists between them. To uncover a reciprocal relationship and fully understand the dynamics of these mechanisms there is a need for longitudinal and qualitative-oriented studies. Second, the measure of self-efficacy is overly simplified in this study because the concept is measured using only three response categories. It is therefore difficult to have a nuanced interpretation of how teachers rate their own self-efficacy. Third, in structural modeling, all variables are run simultaneously, and the omitted variables might also have influenced the explored model.

## Concluding Remarks and Further Research

This paper addresses perspectives on teachers’ ICT self-efficacy for instructional purposes. The results reveal a positive association between teachers’ general ICT self-efficacy, ICT self-efficacy for instructional purposes and the use of ICT in teaching practice. One possible conclusion is that the way to develop ICT self-efficacy for instructional purposes is through a more general ICT self-efficacy. Another possible conclusion is that teachers’ use of ICT in teaching practice can be facilitated by their ICT self-efficacy for instructional purposes.

It seems that collegial collaboration is important for understanding the variations in teachers’ use of ICT in teaching practice. This corresponds to our assumptions that the use of ICT in teaching is a collective project, and that collaboration can provide support and make the use of ICT more legitimate.

As mentioned earlier, Norwegian teachers report that they need help to develop their professional digital competence (Organisation for Economic Co-operation and Development [OECD], 2014). One reason could be that ICT is defined as a transversal skill in the curriculum, meaning that digital skills are embedded in competence aims from different curriculum subjects. The findings from this study indicate that emphasizing teachers and student teachers’ general ICT self-efficacy and ICT self-efficacy for instructional purposes could provide ways of preparing teachers to use ICT in their own teaching practice.

## Ethics Statement

This study was carried out in accordance with the demands of The Norwegian Data Protection Authority and the Norwegian Centre for Research Data. The protocol, the written information to the participants and the procedures of informed consent was approved by the Norwegian Centre for Research Data.

## Author Contributions

IH and OH wrote the introduction and theoretical part of the paper. IH and OH developed the research questions and the hypothesis. IH and OH analyzed the data from an international survey. IH and OH developed the result section. IH and OH completed the discussion and the conclusion. A joint work where both have contributed equally.

## Conflict of Interest Statement

The authors declare that the research was conducted in the absence of any commercial or financial relationships that could be construed as a potential conflict of interest.

## Footnotes

1 For more information about the IEA visit their website: www.iea.nl
